# FoxO3 regulates hepatic triglyceride metabolism via modulation of the expression of sterol regulatory-element binding protein 1c

**DOI:** 10.1186/s12944-019-1132-2

**Published:** 2019-11-15

**Authors:** Liu Wang, Xiaopeng Zhu, Xiaoyang Sun, Xinyu Yang, Xinxia Chang, Mingfeng Xia, Yan Lu, Pu Xia, Hongmei Yan, Hua Bian, Xin Gao

**Affiliations:** 1 Department of Endocrinology and Metabolism, Zhongshan Hospital, Fudan University, Shanghai, 200032 China; 2 Fudan Institute for Metabolic Disease, Fudan University, Shanghai, 200032 China

**Keywords:** Nonalcoholic fatty liver disease, NAFLD, Forkhead box class O3, FoxO3, Sterol regulatory element-binding protein1c, SREBP1c

## Abstract

**Background:**

Excessive intrahepatic lipid accumulation is the major characteristic of nonalcoholic fatty liver disease (NAFLD). We sought to identify the mechanisms involved in hepatic triglyceride (TG) homeostasis. Forkhead box class O (FoxO) transcription factors have been shown to play an important role in hepatic metabolism. However, little is known about the effect of FoxO3 on hepatic TG metabolism.

**Methods:**

Liver biopsy samples from patients with NALFD and liver tissues from high glucose and high sucrose (HFHS) fed mice, ob/ob mice and db/db mice were collected for protein and mRNA analysis. HepG2 cells were transfected with small interfering RNA to mediate FoxO3 knockdown, or adenovirus and plasmid to mediate FoxO3 overexpression. FoxO3-cDNA was delivered by adenovirus to the liver of C57BL/6 J male mice on a chow diet or on a high-fat diet, followed by determination of hepatic lipid metabolism. Sterol regulatory element-binding protein 1c (SREBP1c) luciferase reporter gene plasmid was co-transfected into HepG2 cells with FoxO3 overexpression plasmid.

**Results:**

FoxO3 expression was increased in the livers of HFHS mice, ob/ob mice, db/db mice and patients with NAFLD. Knockdown of FoxO3 reduced whereas overexpression of FoxO3 increased cellular TG concentrations in HepG2 cells. FoxO3 gain-of-function caused hepatic TG deposition in C57BL/6 J mice on a chow diet and aggravated hepatic steatosis when fed a high-fat diet. Analysis of the transcripts established the increased expression of genes related to TG synthesis, including SREBP1c, SCD1, FAS, ACC1, GPAM and DGAT2 in mouse liver. Mechanistically, overexpression of FoxO3 stimulated the expression of SREBP1c, whereas knockdown of FoxO3 inhibited the expression of SREBP1c. Luciferase reporter assays showed that SREBP1c regulated the transcriptional activity of the SREBP1c promoter.

**Conclusions:**

FoxO3 promotes the transcriptional activity of the SREBP1c promoter, thus leading to increased TG synthesis and hepatic TG accumulation.

## Introduction

Nonalcoholic fatty liver disease (NAFLD) is the predominant cause of chronic liver disease. The incidence of NAFLD in the world is 25.24%, with a range of 13.5% in Africa to 31.8% in the Middle East [1]. NAFLD is a highly prevalent metabolic disease closely linked to insulin resistance and metabolic syndrome, leading to an increased risk of liver cirrhosis and hepatocellular carcinoma, type 2 diabetes mellitus, cardiovascular diseases, and chronic kidney disease [2]. The pathogenesis of NAFLD has been extensively studied but remains poorly understood. Disturbed lipid homeostasis and an excessive accumulation of triglyceride (TG) and other lipid species is the first step in the pathophysiology of NAFLD. Insulin resistance, enhanced de novo lipogenesis (DNL), and a high-fat diet are pivotal for the development of hepatic steatosis [3, 4].

Forkhead box class O (FoxO) is a nuclear protein subfamily that includes four homologous proteins in mammals: FoxO1, FoxO3, FoxO4 and FoxO6. These proteins share a highly conserved Forkhead DNA binding domain [5]. FoxOs mediate the inhibitory actions of insulin or insulin-like growth factor on key genes in diverse pathways that include cell cycle regulation, energy metabolism, proteostasis, oxidative stress, apoptosis and immunity [5–9]. Current studies characterized FoxO1 as an important regulator of gluconeogenic activity and lipid metabolism [10]. FoxO3 has the highest degree of homology in amino acid sequence with that of FoxO1 [11], in accordance with mild hepatic glucose production [12]. In lipid metabolism, the homolog of FoxO3 in *C. elegans*, DAF-16, enhanced the expression of gene networks involved in lipid synthesis [13]. However, little is known about the role of FoxO3 in lipid metabolism in mammals. Two cell experiments showed that palmitic acid (PA) or stearate treatment upregulated nuclear FoxO3 protein [14, 15]. Consistently, our team found that FoxO3 expression was elevated in the livers of rats fed a high-fat diet via microarray analysis and confirmed by real-time PCR (Additional file 1: Figure S1a). In human studies, there was a correlation between FoxO3 gene polymorphism and the insulin resistance index and body mass index [16]. Together, these results imply that abnormal expression of FoxO3 may be closely associated with NAFLD. Nonetheless, whether and how FoxO3 affects hepatic TG metabolism has been poorly explored.

DNL is thought to play a significant role in the pathogenesis of NAFLD, with sterol regulatory-element binding protein 1c (SREBP1c) acting as a major regulator [17]. In the context of DNL, SREBP1c is activated to promote the transcription of lipogenic genes such as fatty acid synthase (FAS), acetyl CoA carboxylase 1 (ACC1) and stearoyl-CoA desaturase 1 (SCD1) [18]. Here, we used two independent approaches (loss or gain-of-function) to study what and how FoxO3 exerts effects on hepatic TG metabolism in vitro and in vivo. FoxO3 knockdown suppressed whereas FoxO3 overexpression increased intracellular TG levels. A dual luciferase reporter assay showed that FoxO3 activated the SREBP1c promoter. Thus, we identified that FoxO3 prompted hepatic steatosis via transcriptionally upregulating the expression of SREBP1c.

## Methods

### Human liver samples

We collected eight liver biopsy samples at Zhongshan Hospital, Fudan University. There were four samples from normal subjects and four samples from patients with NAFLD diagnosed by liver biopsy. This study was in accordance with the Helsinki Declaration of 1975 and approved by the ethics committee of Zhongshan Hospital, Fudan University, and each subject provided written informed consent.

### Reagents and antibodies

We obtained insulin, glucose and palmitic acid from Sigma-Aldrich. The anti-FoxO3 antibody (#2497) was purchased from Cell Signaling Technology (Cell Signaling, USA). The anti-SREBP-1 antibody (#8984) was purchased from Santa Cruz Biotechnology, Inc. (Santa Cruz, USA). The mouse monoclonal antibody against β-actin was purchased from Sigma (St. Louis). The goat polyclonal secondary antibodies against mouse (#3032) or rabbit (#3012) were purchased from Signal way Antibody (SAB, USA). The dual-luciferase reporter assay kit was purchased from Promega (Madison, USA).

### Cell culture

Human HepG2 and 293 T cells were maintained in DMEM (Gibco, USA) containing 1% penicillin/streptomycin (Invitrogen, USA), 1 g/liter glucose, and 10% fetal bovine serum (Gibco). For the cell experiments, the cells were cultured in serum free media containing 30 mM glucose and 100 μM palmitic acid for 24 h.

### Cell transfection

For knockdown experiments, three sequences of small interfering RNA (siRNA) targeting human FoxO3 or control non-silencing siRNA (Gene Pharma, China) were transfected into HepG2 cells for 48 h. For overexpression experiments, adenovirus carrying green fluorescent protein (GFP) (Ad-GV314-GFP, GeneChem, China) or FoxO3 coding sequence (Ad-GV314-FoxO3, GeneChem, China) was diluted in PBS and added to media according to the multiplicity of infection (MOI). Generally, HepG2 cells were infected with adenovirus at an MOI of 50 for 24 h. HepG2 cells were transfected with a plasmid expressing green fluorescent protein (GFP), or human FoxO3 (FoxO3-WT, #1787, Addgene, USA), or human FoxO3 with three mutated phosphorylation sites (T32, S253, and S315) (FoxO3-TM, #1788, Addgene, USA).

### Oil red O staining

The cells were washed with PBS twice and fixed with 10% paraformaldehyde for 30 min. Oil red O staining was performed using an Oil red O staining kit (#D027, Jiancheng Biotech, China) according to the manufacturer’s protocols.

### Cellular triglyceride measurement

Cells in 6-well plates were washed twice with PBS, and collected by scraping the cells. Cellular TGs were extracted using the chloroform/methanol (2:1 v/v) method and dried in a chemical hood. After drying, 40 ul 1% Triton X-100-ethol was added [19]. Then, the concentration of TG was measured using a TG reagent kit (Shensuo UNF, China) according to the manufacturer’s protocols.

### Total RNA isolation and quantitative real-time PCR

The total RNA of the cells and liver tissue were isolated using the TRIzol method. An RT reagent kit with cDNA eraser (Takara, Japan) was used to perform reverse transcription. SYBR Green Premix Ex Taq (Takara, Japan) was used to perform quantitative real-time PCR. The data were analyzed by the 2^-△△CT^ method. β-Actin was used as an internal reference. The primers are described in Table 1.

**Table 1:** Sequence of primers for quantitative real-time PCR

Gene	Species	Forward primer	Reverse primer
FoxO3	Human	TCAAGGATAAGGGCGACAGC	GGACCCGCATGAATCGACTA
SREBP-1c	Human	GCGCCTTGACAGGTGAAGTC	GCCAGGGAAGTCACTGTCTTG
SCD1	Human	AGCTCATCGTCTGTGGAGCC	GCCACGTCGGGAATTATGAGG
FAS	Human	GGGATGAACCAGACTGCGTG	TCTGCACTTGGTATTCTGGGT
ACC1	Human	ATGTCTGGCTTGCACCTAGTA	CCCCAAAGCGAGTAACAAATTCT
CD36	Human	CTTTGGCTTAATGAGACTGGGAC	GCAACAAACATCACCACACCA
MTTP	Human	ACAAGCTCACGTACTCCACTG	TCCTCCATAGTAAGGCCACATC
PPARα	Human	TTCGCAATCCATCGGCGAG	CCACAGGATAAGTCACCGAGG
β-actin	Human	GATGAGATTGGCATGGCTTT	GTCACCTTCACCGTTCCAGT
FoxO3	Mouse	CTCACTTTGTCCCAGATCTACG	CTTCATTCTGAACGCGCATG
SREBP-1c	Mouse	GTGAGCCTGACAAGCAATCA	GGTGCCTACAGAGCAAGAG
ChREBP	Mouse	AGATGGAGAACCGACGTATCA	ACTGAGCGTGCTGACAAGTC
SCD1	Mouse	TTCTTGCGATACACTCTGGTGC	CGGGATTGAATGTTCTTGTCGT
FAS	Mouse	GCTGCGGAAACTTCAGGAAAT	AGAGACGTGTCACTCCTGGACTT
ACC1	Mouse	ATGGGCGGAATGGTCTCTTTC	TGGGGACCTTGTCTTCATCAT
GPAM	Mouse	ACAGTTGGCACAATAGACGTTT	CCTTCCATTTCAGTGTTGCAGA
DGAT2	Mouse	GCGCTACTTCCGAGACTACTT	GGGCCTTATGCCAGGAAACT
SREBP-2	Mouse	GCAGCAACGGGACCATTCT	CCCCATGACTAAGTCCTTCAACT
HMGCS1	Mouse	AACTGGTGCAGAAATCTCTAGC	GGTTGAATAGCTCAGAACTAGCC
HMGCR	Mouse	AGCTTGCCCGAATTGTATGTG	TCTGTTGTGAACCATGTGACTTC
36B4	Mouse	AGATTCGGGATATGCTGTTGGC	TCGGGTCCTAGACCAGTGTTC

### Western blot analysis

Protein extraction of cells and liver tissue was performed with RIPA in the presence of protease inhibitors. A BCA-100 Protein Quantitative Analysis Kit (Beyotime Biotechnology, China) was used to measure the protein concentration. A total of 20 μg of protein lysates was loaded onto 10% SDS-PAGE gels and transferred to polyvinylidene difluoride (PVDF) membranes (Millipore) and subjected to immunoblot analysis using antibodies against proteins.

### Dual luciferase reporter assay

A luciferase reporter plasmid encoding the SREBP1c promoter of − 2000/+ 194 was constructed by the GeneChem Corporation (GeneChem, China). HepG2 cells were co-transfected with 0.5 μg of empty vector or FoxO3 plasmid, along with 0.5 μg of luciferase reporter plasmid and 50 ng of pRL-CMV Renilla luciferase plasmid (Promega, USA) as an internal control in 12-well plates via lipo-3000 (Invitrogen) according to the manufacturer’s instructions. After 48 h, dual luciferase reporter assays were performed and analyzed according to the manufacturer’s protocol.

### Animal experiments

We used 9-week-old C57BL/6 J male mice (Shanghai SLAC Laboratory Animal Company) for the chow diet study. We delivered recombinant adenovirus encoding FoxO3-cDNA or control adenovirus by tail vein injection in a total volume of 200 μl. The total viral load was approximately 3 × 10^9^ plaque forming units per mouse. Each animal had free access to drinking water and chow and was housed at constant room temperature (20 ± 2) °C under a 12-h light/dark cycle. All animal experiments were approved by the Animal Use and Care Committee of Fudan University.

We performed the intraperitoneal glucose tolerance tests (IPGTT) on day 7 post-injection and intraperitoneal insulin tolerance tests (IPITT) on day 12 post-injection. On day 19 post-injection, mice were sacrificed after fasting overnight. Blood was collected from the eyeball and centrifuged at 3500 rpm for 15 min at 4 °C. For the obtained liver tissue, some of it was fixed in 10% formaldehyde and analyzed by H&E staining, part was frozen with Tissue OCT-Freeze Medium (Sakura Finetek, USA) for Oil red O staining to evaluate hepatic TG concentration, and the remaining portion was placed in liquid nitrogen for protein, mRNA and TG analysis. Liver H&E staining and Oil red O staining were performed by Wuhan Seville Biotechnology, China. The lipid profiles were detected by Shanghai BioTNT Company.

For the high-fat diet experiment, 8-week-old C57BL/6 J male mice were fed a high-fat diet (D12492, Research Diets, USA) for 20 days, with virus injection at a concentration of 2 × 10^9^ plaque forming units per mouse on day 5. We performed IPGTT on day 7 and IPITT on day 12 post-injection. After day 15 post-injection, mice were sacrificed.

db/db mice, ob/ob mice and high glucose and high sucrose (HFHS) fed mice were gifted by Prof. Yu Li and Prof. Xin Gao.

### Intraperitoneal glucose tolerance tests

Mice were fasted overnight before the experiment and injected intraperitoneally with glucose solution at a dose of 2 g/kg body weight. Blood glucose was measured using a blood glucose meter (ROCHE, Germany) before and 15 min, 30 min, 60 min, and 120 min post-injection.

### Intraperitoneal insulin tolerance tests

Mice were fasted for 4–6 h before the experiment and injected intraperitoneally with regular human insulin at a dose of 1 U/kg body weight. Blood glucose was measured using a blood glucose meter (ROCHE, Germany) before and 15 min, 30 min, 60 min, and 120 min post-infusion.

### Quantification of hepatic lipid content

A total of 20 mg–40 mg of liver tissue was cut and homogenized in 1 ml of PBS. The lipids in the tissue were extracted via the chloroform/methanol (2:1 v/v) method and dried in a chemical hood. After drying, 200 μl of ethanol was added to dissolve it. The concentration of TG was measured using a TG reagent kit (Shensuo UNF, China) according to the manufacturer’s protocols.

### Statistics

All data are presented as the mean ± standard error of the mean (SEM). An unpaired two-tailed t test or Mann-Whitney test was performed for two-group comparisons. For more than two groups, one-way ANOVA was performed for intergroup comparisons. The Chi-square test or Fisher’s exact test was performed for the analysis of contingency tables. All data were analyzed with GraphPad Prism 7. *P* < 0.05 was considered statistically significant.

## Results

### FoxO3 expression is elevated in the liver of NAFLD models and NAFLD patients

**Fig. 1:**
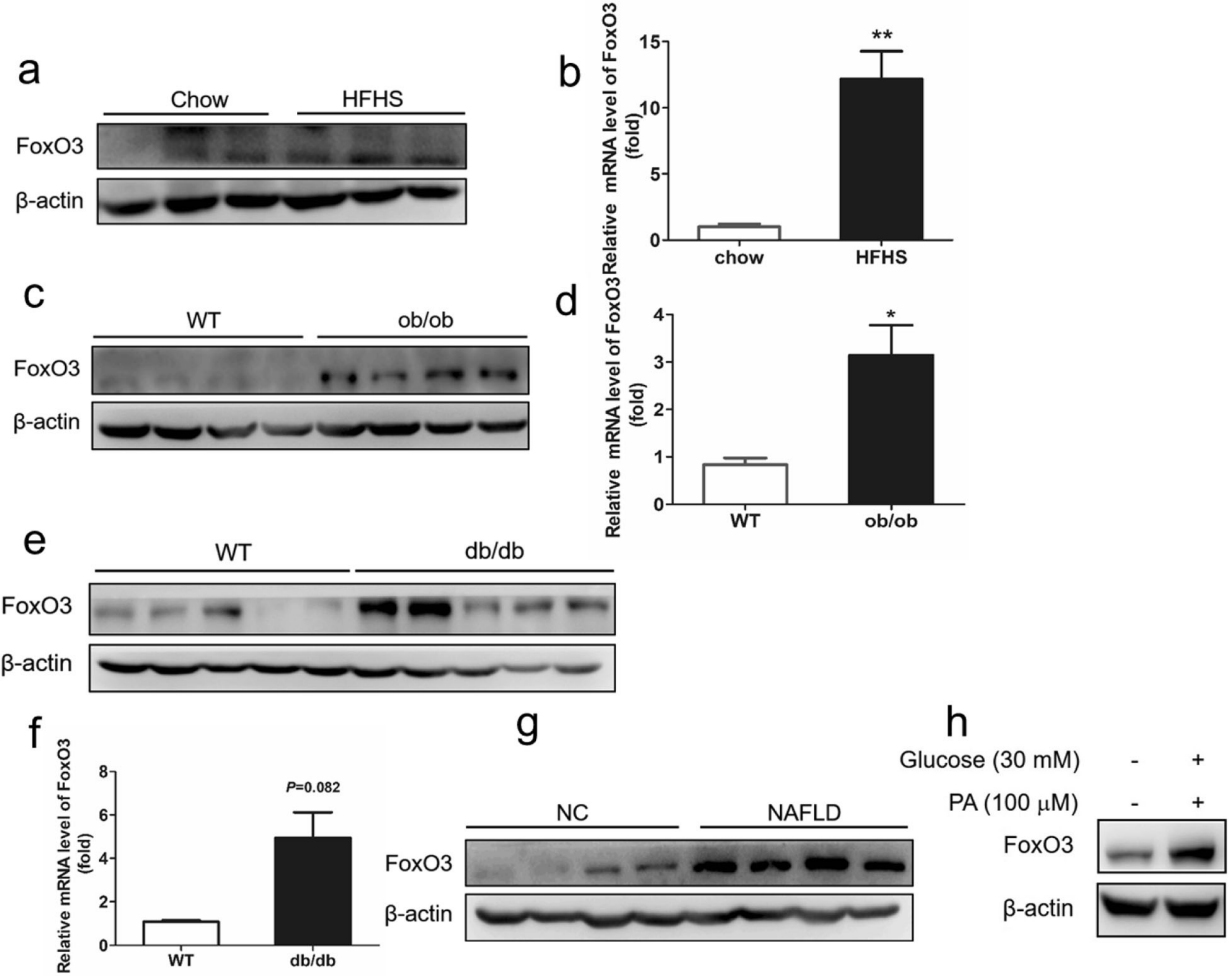
FoxO3 expression is elevated in the liver of NAFLD models and patients with NAFLD. **a** and **b** Protein and mRNA levels of FoxO3 were analyzed in the livers of mice fed a high fat and high sucrose diet (HFHS) or chow diet for 12 weeks. **c** and **d** Protein and mRNA levels of FoxO3 in the livers of ob/ob mice and wild-type (WT) mice fed a chow diet for 12 weeks. **e** and **f** Protein and mRNA levels of FoxO3 in the livers of db/db mice and WT mice fed a chow diet for 12 weeks. **g** The protein level of FoxO3 was measured in the livers of patients with NAFLD and healthy subjects (NC). **h** Protein analysis of FoxO3 in HepG2 cells exposed to high glucose and high palmitic acid (PA) for 24 h. The data are presented as the mean ± SEM, *n* = 3–5 for each group. ^*^P < 0.05 versus control, ^**^P < 0.01 versus control

To determine whether there is a correlation between FoxO3 and NAFLD, we examined the expression of FoxO3 in the liver of different types of NAFLD models. According to the western blotting, the protein level of FoxO3 was upregulated in the livers of high fat and high sucrose (HFHS)-fed mice, ob/ob mice and db/db mice compared with the chow diet mice or wild-type (WT) mice (Fig. 1a, c, e). The mRNA level of FoxO3 was also increased in the livers of HFHS mice and ob/ob mice, of which it was significantly increased in the livers of db/db mice (*P* = 0.082) (Fig. 1b, d, f). Additionally, FoxO3 protein expression was increased dramatically in the livers of NAFLD patients (Fig. 1g). Next, we performed an analysis of FoxO3 protein in vitro. HepG2 cells were exposed to high glucose (30 mM) and high palmitic acid (100 μM, PA) for 24 h to mimic the pathology of NAFLD. Consistently, the protein level of FoxO3 was increased dramatically in HepG2 cells challenged with high glucose and high palmitic acid (HFHG) (Fig. 1h). Additionally, the content of nuclear FoxO3 protein was also increased compared to that of the control group (Additional file 2: Figure S2a). These results indicate that FoxO3 expression becomes abnormally higher in fatty liver and steatotic cells, suggesting a close relationship between NAFLD and FoxO3.

### FoxO3 loss-of-function inhibits triglyceride accumulation in HepG2 cells

**Fig. 2:**
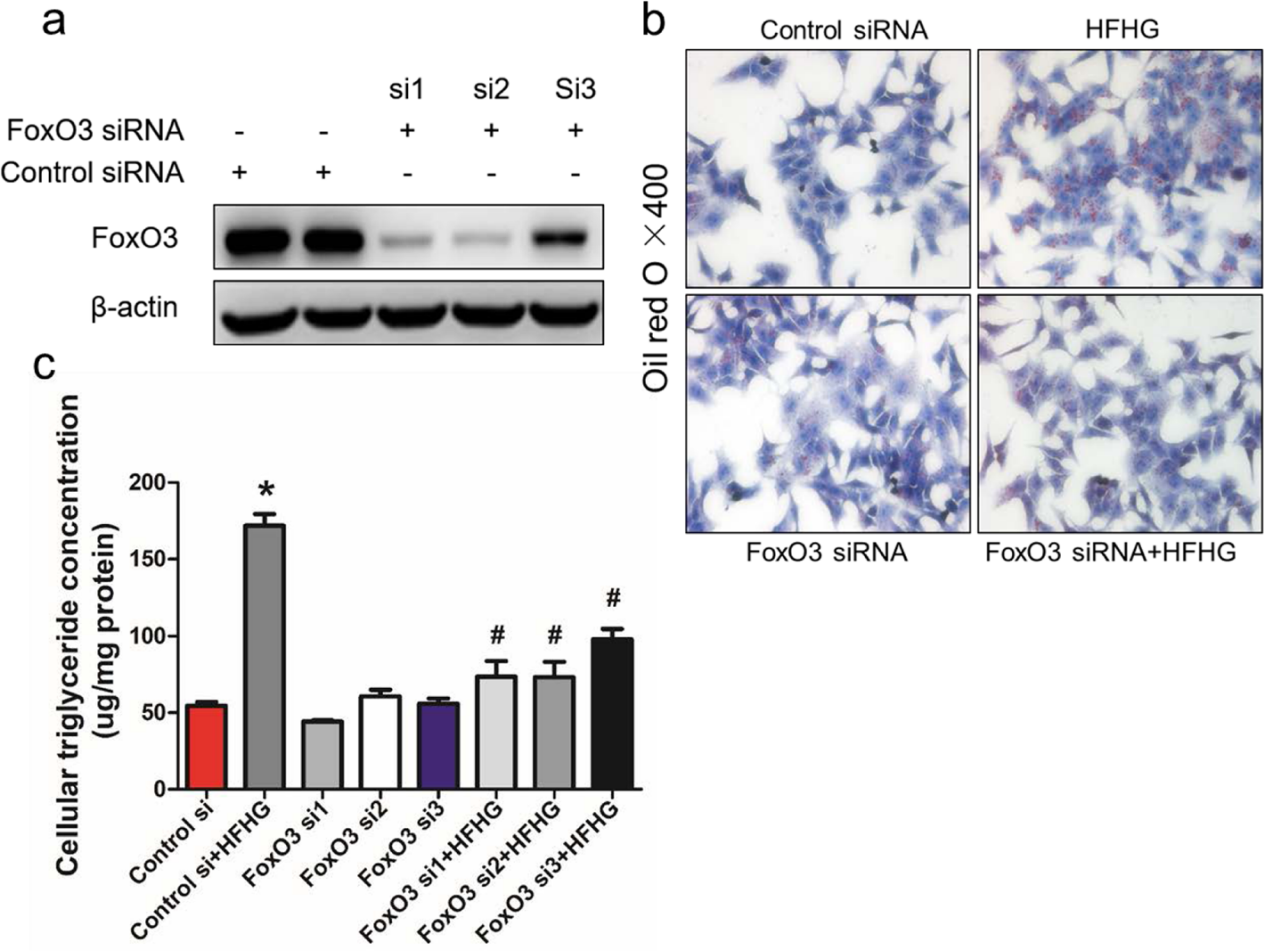
FoxO3 loss-of-function inhibits triglyceride accumulation in HepG2 cells. **a** Protein analysis of FoxO3 in HepG2 cells transduced with three small interfering RNAs (FoxO3 si1, si2, and si3) or a negative control for 24 h. **b** Representative morphology of Oil red O staining of HepG2 cells treated with FoxO3 siRNA1 or negative control for 24 h and then challenged with or without high glucose and high palmitic acid (HFHG) for another 24 h. **c** Analysis of cellular TG concentrations of HepG2 cells transduced with FoxO3 si1, si2, si3 or negative control for 24 h and treated with or without HFHG for another 24 h. The data are presented as the means ± SEM. ^*^P < 0.05 versus control, ^#^*P* < 0.05 versus HFHG

To study the physiological effect of FoxO3 on hepatic TG metabolism, we used siRNA to knock down FoxO3 in HepG2 cells. FoxO3 siRNA1 and siRNA2 transfection triggered a significant decrease in FoxO3 protein levels compared to that of the control group (Fig. 2a). HFHG exposure induced higher levels of lipid droplets in HepG2 cells, which were markedly diminished by FoxO3 siRNA-transfection, as shown by Oil red O staining (Fig. 2b). Quantitatively, the intracellular TG concentration was increased by HFHG administration and was decreased by FoxO3 siRNA treatment (Fig. 2c). These data imply that FoxO3 loss-of-function protects against hepatic steatosis induced by HFHG in vitro.

### FoxO3 gain-of-function results in hepatic steatosis in vitro and in vivo

**Fig. 3:**
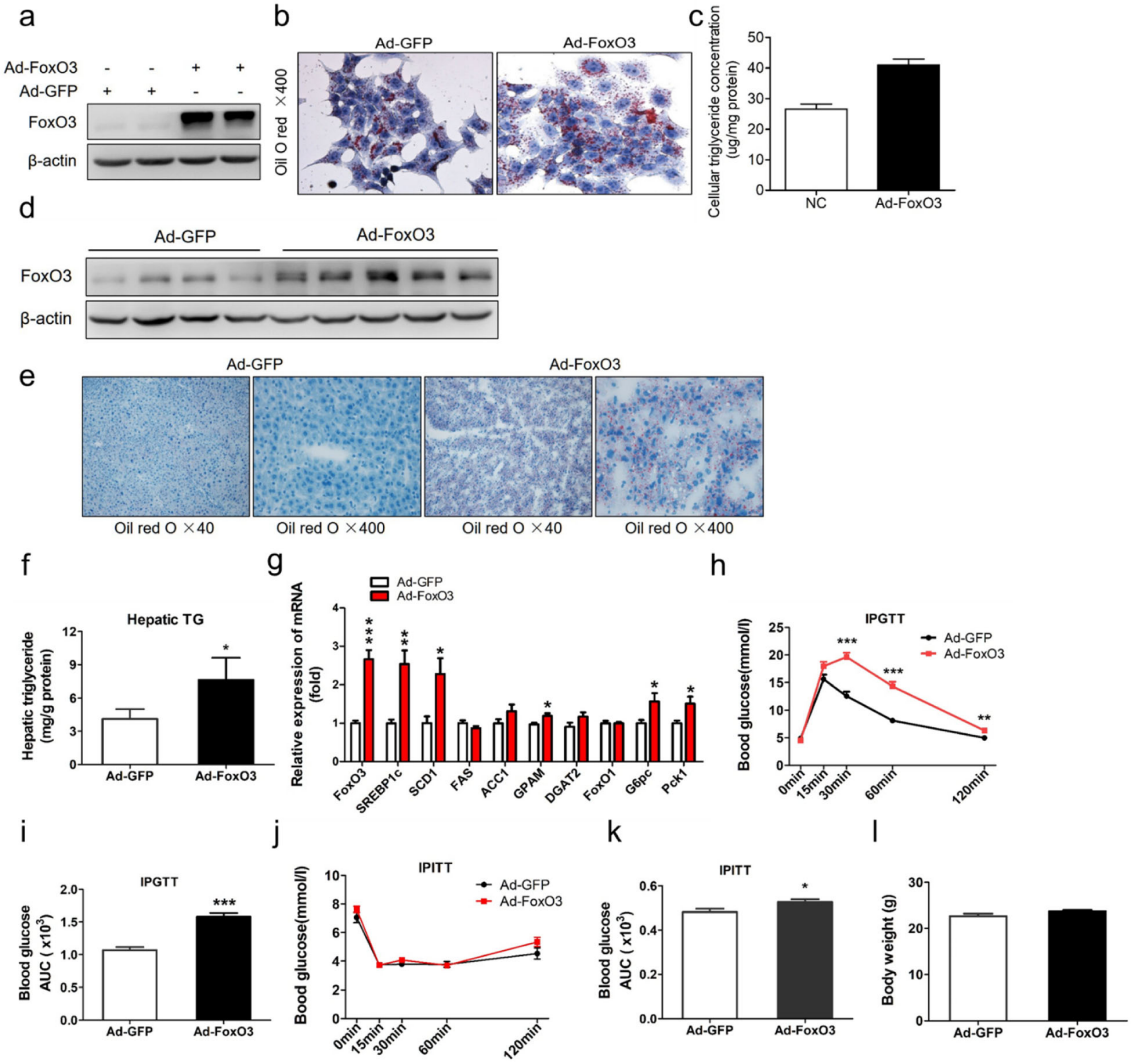
FoxO3 gain-of-function results in hepatic steatosis in vitro and in vivo. **a**-**c** HepG2 cells were infected with GFP -(Ad-GFP)- or FoxO3-overexpressing adenovirus (Ad-FoxO3) at a multiplicity of infection (MOI) of 50 for 24 h. **a** Protein analysis of FoxO3 in the adenovirus infection group and control group. **b** Oil red O staining of HepG2 cells with adenovirus infection. **c** Cellular triglyceride concentration analysis in HepG2 cells treated with adenovirus. **d**-**h** C57BL/6 J male mice (9 weeks old) were injected with Ad-GFP (*n* = 8) or Ad-FoxO3 (*n* = 10) through the tail vein at a concentration of 3 × 10^9^ plaque forming units per mouse, fed a chow diet and were housed under regular light/dark cycles for 19 days. (d) Western blot analysis of liver extracts from adenovirus-injected mice. **e** Oil red O staining of liver sections from representative livers of mice after an overnight fast. **f** Hepatic triglyceride concentration analysis in mice after an overnight fast. **g** Gene expression analysis by RT-PCR in livers from Ad-GFP-treated mice (white bars) or Ad-FoxO3-treated mice (red bars). **h** and **j** Intraperitoneal glucose tolerance tests (IPGTT) and intraperitoneal insulin tolerance tests (IPITT) were performed on day 8 and day 12 respectively after overnight fast. Blood glucose levels were measured in Ad-GFP-treated mice (black line) or Ad-FoxO3-treated mice (red line) before and 15, 30, 60, and 120 min after injection with 2 g/kg dextrose or 1 U/kg insulin intraperitoneally. **i** and **k** Area under the curve (AUC) of blood glucose profiles during IPGTT and IPITT. **l** Body weight analysis of mice. The data are presented as the mean ± SEM. ^*^P < 0.05 versus Ad-GFP; ^**^P < 0.01 versus Ad-GFP, ^***^P < 0.001 versus Ad-GFP. FoxO1, Forkhead box class O 1; SREBP1c, sterol regulatory-element binding protein 1c; SCD1, stearyl-coenzyme A desaturase 1; FAS, fatty acid synthase; ACC1, acetyl-CoA carboxylase 1; GPAM, glycerol-3-phosphate acyltransferase; DGAT2, diacylglycerol acyltransferase 2; G6pc, glucose-6-phosphatase; Pck1, phosphoenolpyruvate carboxykinase

Next, we assessed the role of FoxO3 in TG homeostasis with gain-of-function approaches in the culture of hepatocytes and liver of mice. Overexpression of FoxO3 was achieved by adenovirus infection of HepG2 cells and was confirmed by western blotting (Fig. 3a). FoxO3 overexpression adenovirus (Ad-FoxO3) infection induced increased lipid droplets in HepG2 cells, as shown by Oil red O staining (Fig. 3b). Consistently, cellular TG concentration analysis suggested a trend toward an increase in Ad-FoxO3-treated cells (Fig. 3c). These data indicate a steatosis effect of FoxO3 on hepatocytes.

To confirm the effect of FoxO3 gain-of-function on hepatic steatosis, we injected adenovirus into adult mice via the tail vein. Ad-FoxO3 injection caused an upper shift in FoxO3 protein (Fig. 3d). Histological analysis of liver sections by Oil red O staining showed a high abundance of lipid droplets dispersed in Ad-FoxO3-treated mice compared to Ad-GFP-treated mice (Fig. 3e). The histological result was confirmed by the quantification analysis of hepatic TG content. Ad-FoxO3-treated mice showed 2-fold higher levels of hepatic TG content compared with levels of control littermates (Fig. 3f). To examine what contributed to the higher hepatic TG concentrations, we measured the expression of lipogenic genes. As expected, the liver of Ad-FoxO3-treated mice demonstrated increased gene transcript levels of SREBP1c and its target genes (SCD1) (Fig. 3g). Other genes involved in TG synthesis, such as glycerol-3-phosphate acyltransferase (GPAM), which catalyzes the first committed step of TG synthesis [20], were highly expressed, whereas diacylglycerol acyltransferase 2 (DGAT2), which catalyzes the final committed step of TG synthesis [20], had no significant change (Fig. 3g). FoxO1 has been shown to be an important regulator of lipid metabolism [21]. To determine the potential effect of FoxO3 overexpression on the expression of FoxO1, we detected the mRNA level of FoxO1 and found no significant difference (Fig. 3g).

Carbohydrate response element binding protein (ChREBP), another transcription factor associated with glucose and lipid metabolism [22], was highly expressed in Ad-FoxO3-treated-mice, but did not reach a significant difference (Additional file 3: Figure S3a). Additionally, the mRNA abundance of genes involved in cholesterol synthesis, including sterol regulatory-element binding protein 2 (SREBP-2) [23], 3-hydroxy-3-methylglutaryl-coenzyme A (HMGC) synthase 1 (HMGCS1) and HMGC reductase (HMGCR), had a tendency to increase but only that of SREBP-2 had a significant difference (Additional file 3: Figure S3a).

We also assessed the role of FoxO3 overexpression in glucose metabolism. In response to glucose injection, Ad-FoxO3-treated mice displayed elevated blood glucose levels at 30 min, 60 min and 120 min post-injection, in accordance with an increase in the area under the curve (AUC) (Fig. 3h and i). After insulin injection, the blood glucose concentrations demonstrated no significant difference between the two groups, with a mild increase in the AUC in Ad-FoxO3-treated mice (Fig. 3j and k). Consistently, Ad-FoxO3 treatment increased the gene expression of glucose-6-phosphatase (G6pc) and phosphoenolpyruvate carboxykinase1 (Pck1), two enzymes catalyzing the rate-limiting step of gluconeogenesis (Fig. 3g). No significant difference in body weight was observed (Fig. 3l).

Together, these data indicate that FoxO3 overexpression results in hepatic steatosis and impaired glucose tolerance.

### FoxO3 gain-of-function aggravates hepatic steatosis in mice fed a high-fat diet

**Fig. 4:**
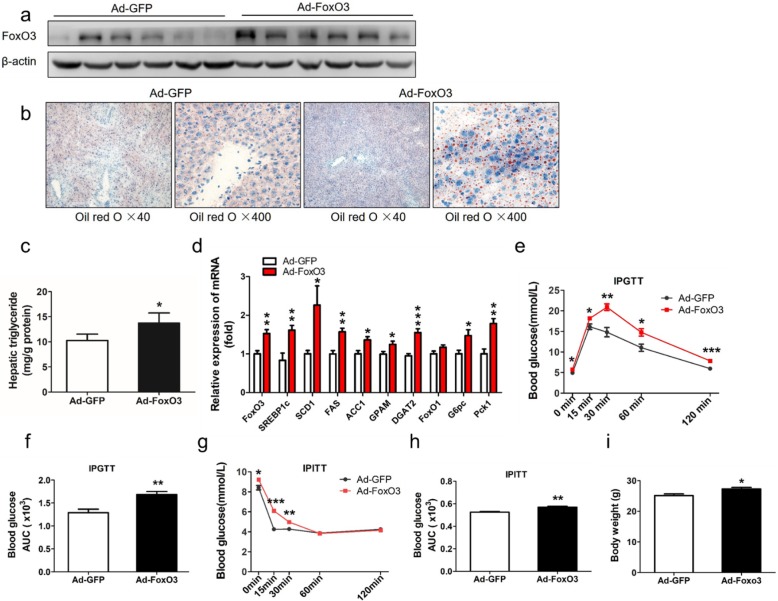
FoxO3 gain-of-function aggravates hepatic steatosis in mice fed a high-fat diet. **a**-**d** C57BL/6 J male mice (8 weeks old) were fed a high-fat diet and were housed under regular light/dark cycles. On day 5, mice were treated with 2 × 10^9^ plaque forming units of Ad-GFP (*n* = 8) or Ad-FoxO3 (*n* = 8) via the tail vein and sacrificed on day 20. **a** Western blot analysis of liver extracts from adenovirus-injected mice. **b** Oil red O staining of liver sections from representative livers of mice after an overnight fast. **c** Intrahepatic triglyceride contents were analyzed in the livers of mice after an overnight fast. **d** Gene expression analysis by RT-PCR in livers from Ad-GFP-treated mice (white bars) or Ad-FoxO3-treated mice (red bars). **e** and **g** Intraperitoneal glucose tolerance tests (IPGTT) and intraperitoneal insulin tolerance tests (IPITT) were performed on day 7 and day 12 respectively after overnight fast. Blood glucose levels were measured in Ad-GFP-treated mice (black line) or Ad-FoxO3-treated mice (red line) before and 15, 30, 60, and 120 min post-injection with 2 g/kg dextrose or 1 U/kg insulin intraperitoneally. **f** and **h** Area under the curve (AUC) of blood glucose profiles during IPGTT and IPITT. **i** Body weight analysis of mice. The data are presented as the mean ± SEM. ^*^P < 0.05 versus Ad-GFP; ^**^P < 0.01 versus Ad-GFP, ^***^P < 0.001 versus Ad-GFP. FoxO1, Forkhead box class O 1; SREBP1c, sterol regulatory-element binding protein 1c; SCD1, stearyl-coenzyme A desaturase 1; FAS, fatty acid synthase; ACC1, acetyl-CoA carboxylase 1; GPAM, glycerol-3-phosphate acyltransferase; DGAT2, diacylglycerol acyltransferase 2; G6pc, glucose-6-phosphatase; Pck1, phosphoenolpyruvate carboxykinase

To assess the contribution of FoxO3 to TG accumulation in the pathological state, we next delivered adenovirus encoding FoxO3-cDNA to mice fed a high-fat diet. Ad-FoxO3-treated-mice showed elevated expression of FoxO3 protein (Fig. 4a). Histologically, Ad-FoxO3-treated mice experienced numerous small and dense lipid droplets, whereas Ad-GFP-treated mice had little lipid droplets (Fig. 4b). Hepatic TG concentrations were also increased in Ad-FoxO3-treated mice (Fig. 4c). Consistently, the mRNA levels of SREBP1c and downstream target genes (SCD1, FAS, and ACC1) were increased, and the expression of genes involved in TG synthesis (GPAM and DGAT2) was increased markedly (Fig. 4d). Consistently, the mRNA expression of FoxO1 showed no significant change (Fig. 4d). The mRNA expression of ChREBP, HMGCS1 and HMGCR was upregulated significantly relative to that of the control (Additional file 3: Figure S3b).

In the glucose tolerance tests, Ad-FoxO3-treated mice exhibited higher levels of blood glucose and increased AUC (Fig. 4e and f). Moreover, insulin tolerance tests revealed that Ad-FoxO3-treated mice showed reduced insulin tolerance at the set point of time of glucose measurements, as determined by an increase in AUC (Fig. 4g and h). Additionally, the mRNA levels of G6pc and Pck1 were also increased (Fig. 4d). Ad-FoxO3-treated mice had more body weight gain than control mice (Fig. 4i).

Together, these data indicate that FoxO3 overexpression aggravates hepatic steatosis and reduces glucose tolerance and insulin sensitivity in mice fed a high-fat diet.

### FoxO3 transcriptionally stimulates the expression of SREBP1c

**Fig. 5:**
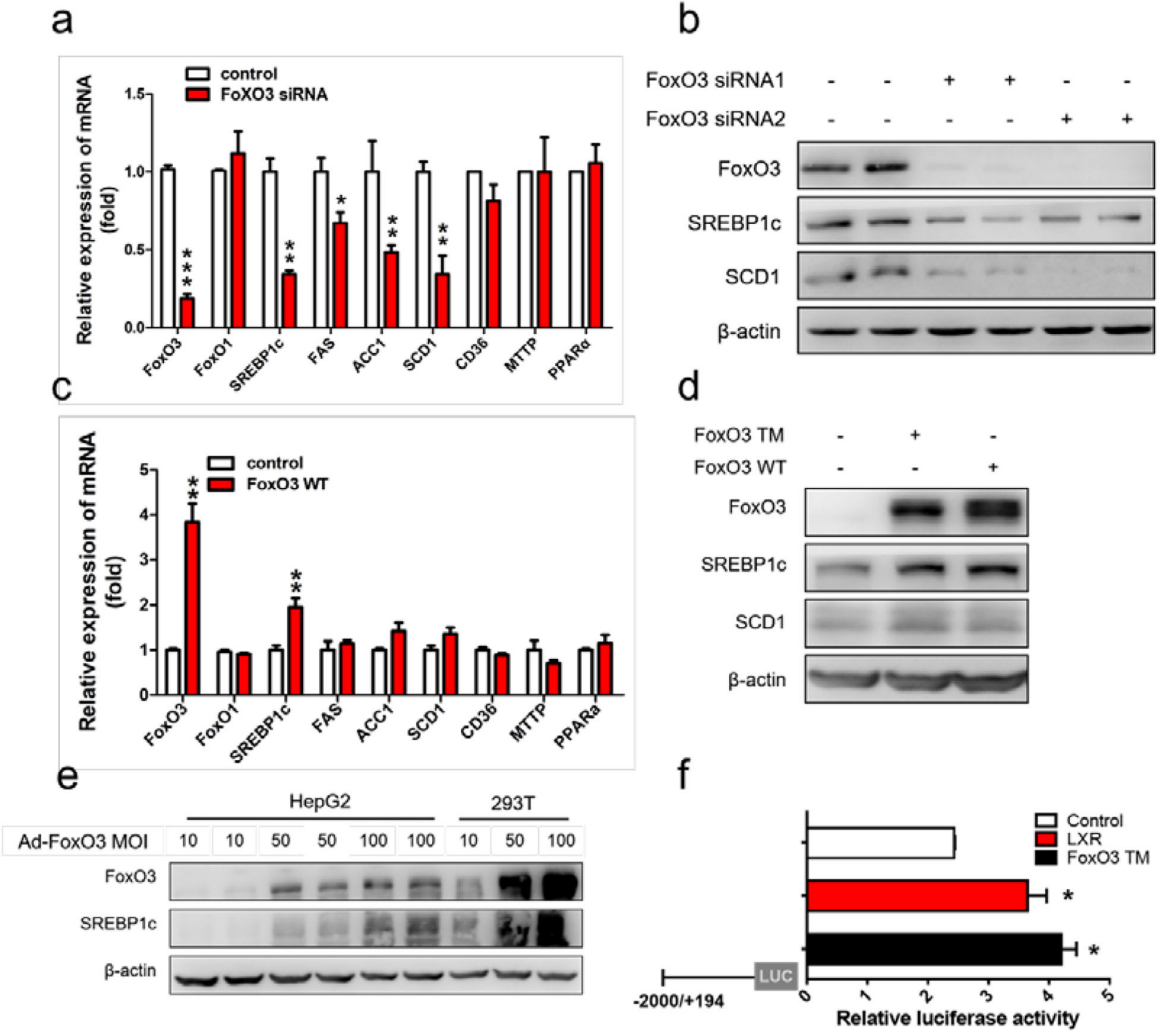
FoxO3 transcriptionally stimulates the expression of SREBP1c. **a** Gene expression analysis of HepG2 cells transduced with FoxO3 siRNA or control siRNA for 48 h. **b** The protein levels of FoxO3, SREBP1c and SCD1 transfected with FoxO3 siRNA or control siRNA. **c** Gene expression analysis of HepG2 cells transfected with plasmid. **d** The protein levels of FoxO3, SREBP1c and SCD1 in HepG2 cells transfected with plasmid. **e** FoxO3 and SREBP1c protein levels of HepG2 cells treated with adenovirus at different multiplicities of infection (MOIs) for 24 h. **f** Luciferase (Luc) reporter assays using constructs of the full-length (− 2000/+ 194) SREBP1c gene promoter of mouse. Luc- SREBP1c was co-transfected into HepG2 cells for 24 h with a plasmid vector expressing the constitutively activated form of FoxO3 (Foxo3-TM) or an empty vector or plasmid expressing LXR. ^*^P < 0.05 versus control, ^**^P < 0.01 versus control, ^***^P < 0.001 versus control. FoxO1, Forkhead box class O 1; SREBP1c, sterol regulatory-element binding protein 1c; SCD1, stearyl-coenzyme A desaturase 1; FAS, fatty acid synthase; ACC1, acetyl-CoA carboxylase 1; CD36, fatty acid translocase protein; MTTP, microsomal triglyceride transfer protein; PPARα, peroxisome proliferator-activated receptor; LXR, liver X factor

To define the underlying mechanism by which FoxO3 functions in hepatic steatosis, we transfected siRNA into HepG2 cells for knockdown of FoxO3 and examined the mRNA levels of genes involved in hepatic TG metabolism. Hepatic TG homeostasis is controlled by at least four pathways including free fatty acid uptake, fatty acid oxidation, de novo lipogenesis (DNL) and TG secretion. Fatty acid transmembrane transporter (CD36), peroxisome proliferator-activated receptor alpha (PPARα), SREBP1c and microsomal triglyceride transfer protein (MTTP) are the key regulators [24]. Interestingly, FoxO3 knockdown curbed the transcription and mRNA levels of SREBP1c and its downstream target genes (SCD1, FAS, and ACC1), without significantly influencing CD36, PPARα and MTTP expression (Fig. 5a). FoxO3 downregulation by siRNA resulted in a marked repression of SREBP1c and SCD1 protein expression (Fig. 5b). FoxO3 overexpression mediated by plasmid increased the mRNA level of SREBP1c, without influencing the mRNA levels of CD36, PPARα and MTTP (Fig. 5c). Plasmid-mediated overexpression of FoxO3 increased the protein levels of SREBP1c and SCD1 (Fig. 5d). Additionally, an induction of SREBP1c, in a manner that paralleled the increase in FoxO3 expression, was observed in HepG2 cells infected with Ad-FoxO3 (Fig. 5e). These observations imply that FoxO3 mediates the expression of SREBP1c.

FoxO3 has been shown to act as a transcriptional activator to regulate target gene expression [25]. To gain additional insight into the molecular mechanisms underlying the induction of SREBP1c by FoxO3, we analyzed the promoter of mouse and human SREBP1c genes and identified a novel FoxO3 binding site using the JASPAR database (http://jaspar.genereg.net/cgi-bin/jaspar_db.pl) (Additional file 4: Figure S4a and b). To confirm this mechanism, we performed a dual-luciferase reporter assay to detect the capacity of FoxO3 to activate the promoter of SREBP1c in HepG2 cells. FoxO3-TM co-transfection resulted in a two-fold induction of luciferase activity, equivalent to the increased activity induced by plasmid expressing LXR (Fig. 5f). These data indicate that FoxO3 transcriptionally activates the activity of the SREBP1c promoter.

## Discussion

In this work, we characterized the role of FoxO3 in hepatic TG deposition in vitro and in vivo. We showed that knockdown or overexpression of FoxO3 reduced or increased lipid droplets in HepG2 cells. In vivo, FoxO3 gain of function increased TG accumulation, along with up-regulation of numerous lipogenic genes (SREBP1c, SCD1, FAS, ACC1, GPAM and DGAT2). Mechanically, FoxO3 promotes the transcriptional activity of the SREBP1c promoter.

In this study, we confirmed the abnormally higher expression of FoxO3 in various steatosis cell models, animal models and patients with NAFLD, implying a close link between FoxO3 and fatty liver. Further, FoxO3 loss of function via siRNA abolished the lipid accumulation induced by HFHG treatment in HepG2 cells, and overexpression of FoxO3 mimicked the effect of HFHG. In vivo, FoxO3 gain of function prompted hepatic TG deposition and impaired glucose tolerance on a chow diet, and those impacts were further enhanced when fed a high-fat diet. These results indicate that FoxO3 stimulates lipid accumulation in vitro and in vivo.

Current studies have highlighted the importance of FoxOs, especially FoxO1, in hepatic lipid metabolism using liver-specific triple-null mice or overexpression models. Combined ablation of FoxOs studies exhibited changes in the hepatic lipid content [12, 26, 27], whereas FoxO1 gain-of-function studies seemed to imply both positive and negative effects on lipid production and accumulation. For overexpression of FoxO1 in vivo, there are two studies showing increased hepatic TG [28, 29], one showing no change [30], and one showing decreased hepatic TG [31]. Together, FoxO1 is involved in liver lipid metabolism but the exact effects are conflicting. To our knowledge, there is only one study about FoxO3 loss or gain of function on hepatic TG deposition [12]. In this study, FoxO3 ablation exerted no impact on liver histopathology, but increased the gene transcriptional level of FAS in vivo. In vitro, FoxO3 gain of function decreased the mRNA level of FAS [12]. In contrast, we detected an increase in FAS mRNA levels in Ad-FoxO3-injected mice. This discrepancy may be due to the differences in the level and model of FoxO3 overexpression, of which in their experiment, it was by 1500-fold mediated by plasmid, and in our study, it was 100-fold mediated by adenovirus. Moreover, the expression of FoxO1 remained unchanged with FoxO3 gain-of-function or knockdown in vitro and in vivo, excluding the interference of FoxO1 on lipid metabolism. Our study is the first to clarify that FoxO3 prompted hepatic TG deposition in vitro and in vivo.

The effect of FoxO3 on cholesterol metabolism has been poorly explored. Only one study reported that FoxO3 and sirt6 reduced LDL-cholesterol levels through regulation of the Pcsk9 gene [32]. Our study showed that hepatic FoxO3 overexpression led to increased expression of cholesterol synthesis genes (SREBP-2, HMGCR, and HMGCS1), indicating a potential effect of FoxO3 on cholesterol homeostasis that is worthy of further investigation.

Ad-FoxO3-treated mice displayed increased blood glucose concentrations after injection of glucose or insulin, indicating the impaired glucose tolerance and reduced insulin tolerance. This is in line with previous reports, showing that FoxO3 has a synergistic effect with FoxO1 on hepatic gluconeogenesis. Hepatic deletion of FoxO1 reduced fasting blood glucose concentrations by 20%, and combined deletion of FoxO1 and FoxO3 further reduced fasting blood glucose concentrations by 40% [12]. Either FoxO1 or FoxO3 gain of function in vitro increased the gene expression of G6pc and Pck1 [12]. Additionally, intrahepatic TG accumulation also impairs insulin sensitivity and causes elevated blood glucose levels [33]. Thus, the reduced glucose tolerance and insulin tolerance in our study are likely due to the upregulated expression of gluconeogenic genes or the indirect effect of hepatic TG accumulation.

Our study points to a transcriptional stimulus of FoxO3 on SREBP1c gene expression to exert effects on liver histopathology. This conclusion is based on the following observations: 1) transfection with Foxo3 siRNA in HepG2 cells lowered the protein and mRNA levels of SREBP1c; 2) adenovirus or plasmid mediated overexpression of FoxO3 resulted in increased expression of SREBP1c; 3) SREBP1c expression was increased in the livers of Ad-FoxO3-treated mice; and 4) the constitutively active form of FoxO3 increased SREBP-1c promoter activity. The FoxO3-SREBP1c pathway is activated in over-nutrition states, as evidenced by the increased nuclear protein levels of FoxO3 and SREBP1c in our study. However, this pathway is inhibited under physiological conditions of inactivation of FoxO3 induced by insulin-dependent phosphorylation. Additionally, the mRNA expression of ChREBP, a glucose-activated transcription factor regulating glycolytic and lipogenic genes [22], was increased in the liver of Ad-FoxO3-treated mice, implying that activated SREBP1c may account partially for the hepatic lipid deposition in this study.

In the luciferase activity experiments, FoxO3-TM increased the Luc-SREBP1c luciferase activity by 2-fold, equivalent to the increased activity observed in the positive control treated with LXR plasmid. LXR plays a key role in the transcriptional activation of SREBP1c [34]. This relatively lower stimulation may stem from the interference of endogenous SREBP1c activity. Endogenous SREBP1c binds to the SRE site on the SREBP1c promoter for feed-forward regulation [35], resulting in a relatively high luciferase activity in the control group. Moreover, a dual luciferase experiment implicates that FoxO3 activates the promoter of SREBP1c, and whether FoxO3 binds directly to the promoter of SREBP1c remains unknown. Studies have reported that there is no known FoxO-binding site in the proximal promoter of the SREBP1c promoter [36]. Moreover, studies showed that FoxO3 modulates gene expression via sequestering transcription factors and co-activators or binding to enhancers: FoxO3 recruits Sirt6 to the proximal promoter region of the proprotein convertase subtilisin/kexin type 9 gene to regulate cholesterol homeostasis [32]; FoxO3 binds to enhancers and increases polymerase II recruitment to active target genes [37]. Thus, we hypothesized that FoxO3 activation of the promoter of SREBP1c may not depend on direct promoter- binding. In conclusion, the underlying mechanism of how FoxO3 prompts SREBP1c gene expression should be investigated in further studies. Moreover, whether SREBP1c is required for the effect of FoxO3 on hepatic steatosis needs to be further explored.

## Conclusions

The present study identifies that FoxO3 prompts hepatic triglyceride accumulation via transcriptionally stimulating the expression of SREBP1c. The FoxO3-SREBP1c pathway may play a potential role in the pathophysiology of NAFLD.

## Supplementary information

**Additional file 1: Figure S1.** FoxO3 expression was elevated in the livers of rats fed a high-fat diet. (a) Hepatic mRNA level of FoxO3. *P < 0.05 vs. chow.

**Additional file 2: Figure S2.** FoxO3 was activated in HepG2 cells exposed to high glucose and high palmitic acid. (a) Nuclear protein of FoxO3 and SREBP1c in HepG2 cells exposed to high glucose and high palmitic (PA).

**Additional file 3: Figure S3.** The expression of ChREBP, SREBP-2, HMGCS1 and HMGCR was changed in the livers of mice injected with Ad-FoxO3. (a) mRNA levels of ChREBP, SREBP-2, HMGCS1 and HMGCR in the livers of mice treated with adenovirus fed a chow diet. (b) mRNA levels of ChREBP, SREBP-2, HMGCS1 and HMGCR in the livers of mice treated with adenovirus fed a high-fat diet (HFD). Ad-GFP, adenovirus expressing GFP; Ad-FoxO3, adenovirus expressing FoxO3. *P < 0.05 vs. Ad-GFP, **P < 0.01 vs. Ad-GFP.

**Additional file 4: Figure S4.** FoxO3 binding sites may exist in the promoter of SREBP1c. (a) The predicted FoxO3 binding sites in the promoter of the mouse SREBP1c gene using the JASPAR database. (b) The predicted FoxO3 binding sites in the promoter of the human SREBP1c gene using the JASPAR database.

## Data Availability

All the data generated or analyzed during this study are included in this published article.

## Abbreviations

ACC1: Acetyl CoA carboxylase 1
Ad-FoxO3: FoxO3-overexpressing adenovirus
Ad-GFP: GFP-overexpressing adenovirus
AUC: Area under the curve
CD36: Fatty acid transmembrane transporter
ChREBP: Carbohydrate response element binding protein
DGAT2: Diacylglycerol acyltransferase 2
DNL: De novo lipogenesis
FAS: Fatty acid synthase
FoxO: Forkhead box class O
FoxO3-TM: Plasmid vector expressing the constitutively activated form of FoxO3
FoxO3-WT: Plasmid vector expressing FoxO3
G6pc: Glucose-6-phosphatase
GPAM: Glycerol-3-phosphate acyltransferase
HFHG: High glucose and high palmitic acid
HMGCR: HMGC reductase
HMGCS1: 3-hydroxy-3-methylglutaryl-coenzyme A (HMGC) synthase 1
IPGTT: Intraperitoneal glucose tolerance tests
IPITT: Intraperitoneal insulin tolerance tests
LXR: Liver X receptor
MOI: Multiplicity of infection
mRNA: Messenger RNA
MTTP: Microsomal triglyceride transfer protein
NAFLD: Nonalcoholic fatty liver disease
PA: Palmitic acid
Pck1: Phosphoenolpyruvate carboxykinase
PPARα: Peroxisome proliferator-activated receptor alpha
PVDF: Polyvinylidene difluoride
RT-PCR: Real-time polymerase chain reaction
SCD1: Stearyl-coenzyme A desaturase 1
SEM: Standard error of the mean
siRNA: Small interfering RNA
SRE: Sterol regulatory element
SREBP1c: Sterol-regulatory element-binding protein1c
SREBP-2: Sterol regulatory-element binding protein 2
TG: Triglyceride

## References

[1] Younossi Z, Anstee QM, Marietti M, Hardy T, Henry L, Eslam M (2017). Global burden of NAFLD and NASH: trends, predictions, risk factors and prevention. Nat Rev Gastro Hepat.

[2] Byrne CD, Targher G (2014). NAFLD: a multisystem disease. J Hepatol.

[3] Hardy T, Oakley F, Anstee QM, Day CP (2016). Nonalcoholic fatty liver disease: pathogenesis and disease Spectrum. Annu Rev Pathol Mech Dis.

[4] Lambert JE, Ramos-Roman MA, Browning JD, Parks EJ (2014). Increased De novo Lipogenesis is a distinct characteristic of individuals with nonalcoholic fatty liver disease. Gastroenterology.

[5] Brent MM, Anand R, Marmorstein R (2008). Structural basis for DNA recognition by FoxO1 and its regulation by posttranslational modification. Structure.

[6] Tia N, Singh AK, Pandey P, Azad CS, Chaudhary P, Gambhir IS (2018). Role of Forkhead box O (FOXO) transcription factor in aging and diseases. Gene.

[7] Kousteni S (2012). FoxO1, the transcriptional chief of staff of energy metabolism. Bone.

[8] Calnan DR, Brunet A (2008). The FoxO code. Oncogene.

[9] Webb AE, Brunet A (2014). FOXO transcription factors: key regulators of cellular quality control. Trends Biochem Sci.

[10] Lee S, Dong HH (2017). FoxO integration of insulin signaling with glucose and lipid metabolism. J Endocrinol.

[11] Tzivion G, Dobson M, Ramakrishnan G (1813). FoxO transcription factors; regulation by AKT and 14-3-3 proteins. Biochim Biophys Acta.

[12] Zhang K, Li L, Qi Y, Zhu X, Gan B, DePinho RA (2012). Hepatic suppression of Foxo1 and Foxo3 causes hypoglycemia and hyperlipidemia in mice. Endocrinology.

[13] Amrit FRG, Steenkiste EM, Ratnappan R, Chen S, McClendon TB, Kostka D (2016). DAF-16 and TCER-1 facilitate adaptation to Germline loss by restoring lipid homeostasis and repressing reproductive physiology in C. elegans. PLoS Genet.

[14] Natarajan SK, Ingham SA, Mohr AM, Wehrkamp CJ, Ray A, Roy S (2014). Saturated free fatty acids induce cholangiocyte lipoapoptosis. Hepatology.

[15] Barreyro FJ, Kobayashi S, Bronk SF, Werneburg NW, Malhi H, Gores GJ (2007). Transcriptional regulation of Bim by FoxO3A mediates hepatocyte Lipoapoptosis. J Biol Chem.

[16] Ragab S, Abdallah N, Hasan NS, Kandil ME, El Wasseif M, Elhosary Y (2014). FOXO 1a and FOXO 3a gene polymorphisms in association with metabolic syndrome. J Genet Eng Biotechnol.

[17] Ferré P, Foufelle F (2010). Hepatic steatosis: a role for de novo lipogenesis and the transcription factor SREBP-1c. Diabetes Obes Metab.

[18] Sanders FW, Griffin JL (2016). De novo lipogenesis in the liver in health and disease: more than just a shunting yard for glucose. Biol Rev Camb Philos Soc.

[19] Zhu X, Yan H, Xia M, Chang X, Xu X, Wang L, et al. Metformin attenuates triglyceride accumulation in HepG2 cells through decreasing stearyl-coenzyme a desaturase 1 expression. Lipids Health Dis. 2018;17. 10.1186/s12944-018-0762-0 PMC5952420 29759071

[20] Wang H, Airola MV, Reue K (1862). How lipid droplets "TAG" along: Glycerolipid synthetic enzymes and lipid storage. Biochim Biophys Acta Mol Cell Biol Lipids.

[21] Li Y, Ma Z, Jiang S, Hu W, Li T, Di S (2017). A global perspective on FOXO1 in lipid metabolism and lipid-related diseases. Prog Lipid Res.

[22] Xu X, So JS, Park JG, Lee AH (2013). Transcriptional control of hepatic lipid metabolism by SREBP and ChREBP. Semin Liver Dis.

[23] Eberle D, Hegarty B, Bossard P, Ferre P, Foufelle F (2004). SREBP transcription factors: master regulators of lipid homeostasis. Biochimie.

[24] Kawano Y, Cohen DE (2013). Mechanisms of hepatic triglyceride accumulation in non-alcoholic fatty liver disease. J Gastroenterol.

[25] Eijkelenboom A, Mokry M, de Wit E, Smits LM, Polderman PE, van Triest MH (2013). Genome-wide analysis of FOXO3 mediated transcription regulation through RNA polymerase II profiling. Mol Syst Biol.

[26] Haeusler RA, Hartil K, Vaitheesvaran B, Arrieta-Cruz I, Knight CM, Cook JR, et al. Integrated control of hepatic lipogenesis versus glucose production requires FoxO transcription factors. Nat Commun. 2014;5190. 10.1038/ncomms6190 PMC4197140 25307742

[27] Tao Rongya, Wei Dan, Gao Hanlin, Liu Yunlong, DePinho Ronald A., Dong X. Charlie (2011). Hepatic FoxOs Regulate Lipid Metabolism via Modulation of Expression of the Nicotinamide Phosphoribosyltransferase Gene. Journal of Biological Chemistry.

[28] Matsumoto M (2006). Dual role of transcription factor FoxO1 in controlling hepatic insulin sensitivity and lipid metabolism. J Clin Invest.

[29] Qu S, Altomonte J, Perdomo G, He J, Fan Y, Kamagate A (2006). Aberrant forkhead box O1 function is associated with impaired hepatic metabolism. Endocrinology.

[30] Zhang W, Patil S, Chauhan B, Guo S, Powell DR, Le J (2006). FoxO1 regulates multiple metabolic pathways in the liver. J Biol Chem.

[31] Zhang W, Bu SY, Mashek MT, O-Sullivan I, Sibai Z, Khan SA (2016). Integrated regulation of hepatic lipid and glucose metabolism by adipose triacylglycerol lipase and FoxO proteins. Cell Rep.

[32] Tao R, Xiong X, DePinho RA, Deng C, Dong XC (2013). FoxO3 transcription factor and Sirt6 Deacetylase regulate low density lipoprotein (LDL)-cholesterol homeostasis via control of the Proprotein Convertase Subtilisin/Kexin type 9 (Pcsk9) gene expression. J Biol Chem.

[33] Farese RV, Zechner R, Newgard CB, Walther TC (2012). The problem of establishing relationships between hepatic Steatosis and hepatic insulin resistance. Cell Metab.

[34] Chen GX, Liang GS, Ou JF, Goldstein JL, Brown MS (2004). Central role for liver X receptor in insulin-mediated activation of Srebp-1c transcription and stimulation of fatty acid synthesis in liver. P Natl Acad Sci Usa.

[35] Amemiya-Kudo M, Shimano H, Yoshikawa T, Yahagi N, Hasty AH, Okazaki H (2000). Promoter analysis of the mouse sterol regulatory element-binding protein-1c gene. J Biol Chem.

[36] Deng X, Zhang W, O-Sullivan I, Williams JB, Dong Q, Park EA (2012). FoxO1 inhibits sterol regulatory element-binding protein-1c (SREBP-1c) gene expression via transcription factors Sp1 and SREBP-1c. J Biol Chem.

[37] Eijkelenboom A, Mokry M, Smits LM, Nieuwenhuis EE, Burgering BMT (2013). FOXO3 selectively amplifies enhancer activity to establish target gene regulation. Cell Rep.

