# Supplementary figures and images for: FoxO3 regulates hepatic triglyceride metabolism via modulation of the expression of sterol regulatory-element binding protein 1c
Source: Lipids Health Dis. 2019 Nov 15;18:197. doi: 10.1186/s12944-019-1132-2 (PMC6857156; doi:10.1186/s12944-019-1132-2)

**Supplemental** **Figure 1**

**
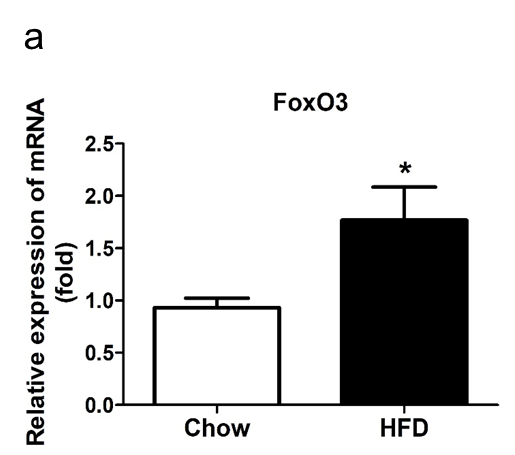
**

Supplement: Supplementary file 1 — Additional file 1: Figure S1. FoxO3 expression was elevated in the livers of rats fed a high-fat diet. (a) Hepatic mRNA level of FoxO3. *P < 0.05 vs. chow. [file 12944_2019_1132_MOESM1_ESM.docx]

**Supplemental** **Figure 2**


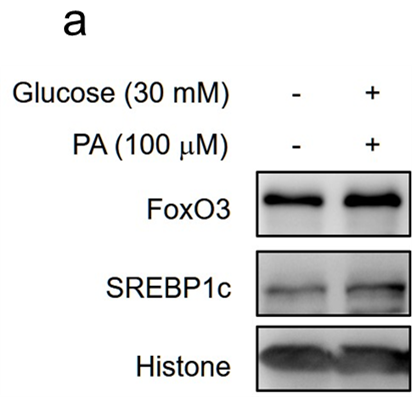

Supplement: Supplementary file 2 — Additional file 2: Figure S2. FoxO3 was activated in HepG2 cells exposed to high glucose and high palmitic acid. (a) Nuclear protein of FoxO3 and SREBP1c in HepG2 cells exposed to high glucose and high palmitic (PA). [file 12944_2019_1132_MOESM2_ESM.docx]

**Supplemental** **Figure 3**

**
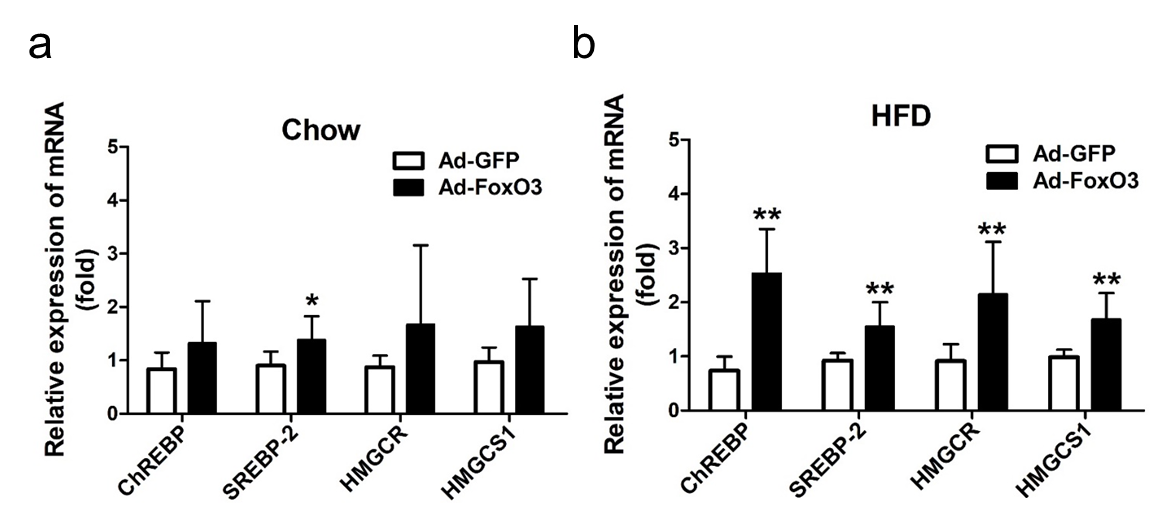
**

Supplement: Supplementary file 3 — Additional file 3: Figure S3. The expression of ChREBP, SREBP-2, HMGCS1 and HMGCR was changed in the livers of mice injected with Ad-FoxO3. (a) mRNA levels of ChREBP, SREBP-2, HMGCS1 and HMGCR in the livers of mice treated with adenovirus fed a chow diet. (b) mRNA levels of ChREBP, SREBP-2, HMGCS1 and HMGCR in the livers of mice treated with adenovirus fed a high-fat diet (HFD). Ad-GFP, adenovirus expressing GFP; Ad-FoxO3, adenovirus expressing FoxO3. *P < 0.05 vs. Ad-GFP, **P < 0.01 vs. Ad-GFP. [file 12944_2019_1132_MOESM3_ESM.docx]

**Supplemental** **Figure 4**

**
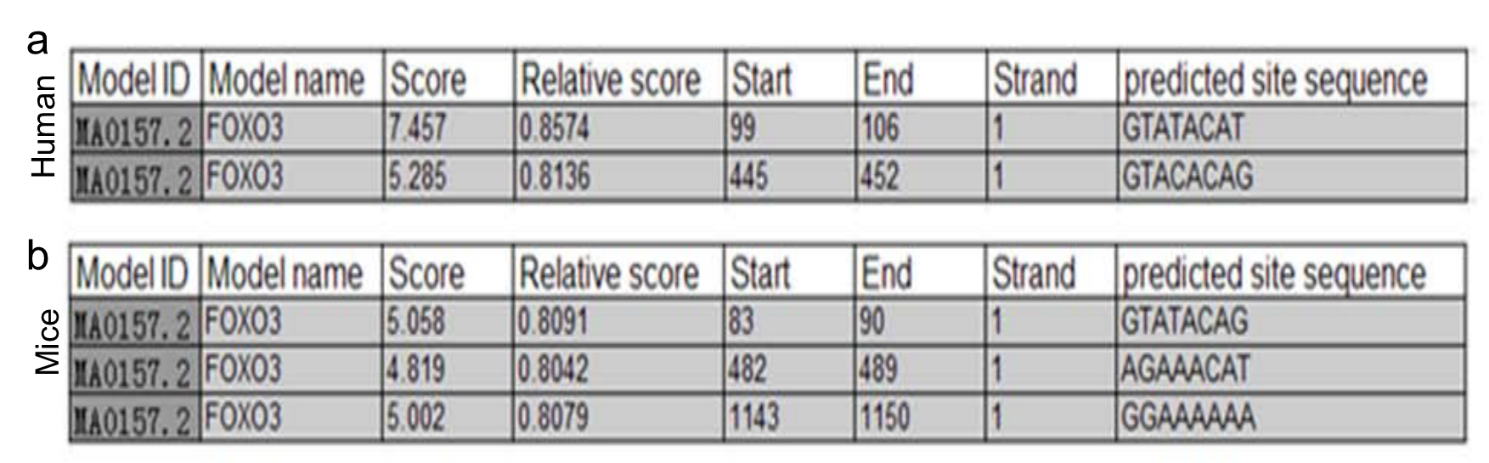
**

Supplement: Supplementary file 4 — Additional file 4: Figure S4. FoxO3 binding sites may exist in the promoter of SREBP1c. (a) The predicted FoxO3 binding sites in the promoter of the mouse SREBP1c gene using the JASPAR database. (b) The predicted FoxO3 binding sites in the promoter of the human SREBP1c gene using the JASPAR database. [file 12944_2019_1132_MOESM4_ESM.docx]
